# Development and Validation of a Sociodemographic and Behavioral Characteristics-Based Risk-Score Algorithm for Targeting HIV Testing Among Adults in Kenya

**DOI:** 10.1007/s10461-020-02962-7

**Published:** 2020-07-10

**Authors:** Hellen Muttai, Bernard Guyah, Paul Musingila, Thomas Achia, Fredrick Miruka, Stella Wanjohi, Caroline Dande, Polycarp Musee, Fillet Lugalia, Dickens Onyango, Eunice Kinywa, Gordon Okomo, Iscah Moth, Samuel Omondi, Caren Ayieko, Lucy Nganga, Rachael H. Joseph, Emily Zielinski-Gutierrez

**Affiliations:** 1 Division of Global HIV & TB (DGHT), United States Centers for Disease Control and Prevention (CDC), Kenya, KEMRI Campus, P.O. Box 606, Nairobi, 00621 Kenya; 2 School of Public Health, Maseno University, Kisumu, Kenya; 3 Center for Health Solution, Kisumu, Kenya; 4 University of California at San Francisco, Kisumu, Kenya; 5 Elizabeth Glaser Pediatric AIDS Foundation, Homa Bay, Kenya; 6 Columbia University, Kisumu, Kenya; 7 Kisumu County Department of Health, Kisumu, Kenya; 8 Homa Bay County Department of Health, Homa Bay, Kenya; 9 Siaya County Department of Health, Siaya, Kenya

**Keywords:** HIV testing, Risk-score algorithm, Kenya

## Abstract

To inform targeted HIV testing, we developed and externally validated a risk-score algorithm that incorporated behavioral characteristics. Outpatient data from five health facilities in western Kenya, comprising 19,458 adults ≥ 15 years tested for HIV from September 2017 to May 2018, were included in univariable and multivariable analyses used for algorithm development. Data for 11,330 adults attending one high-volume facility were used for validation. Using the final algorithm, patients were grouped into four risk-score categories: ≤ 9, 10–15, 16–29 and ≥ 30, with increasing HIV prevalence of 0.6% [95% confidence interval (CI) 0.46–0.75], 1.35% (95% CI 0.85–1.84), 2.65% (95% CI 1.8–3.51), and 15.15% (95% CI 9.03–21.27), respectively. The algorithm’s discrimination performance was modest, with an area under the receiver-operating-curve of 0.69 (95% CI 0.53–0.84). In settings where universal testing is not feasible, a risk-score algorithm can identify sub-populations with higher HIV-risk to be prioritized for HIV testing.

**Electronic supplementary material:**

The online version of this article (10.1007/s10461-020-02962-7) contains supplementary material, which is available to authorized users.

## Introduction

With the global commitment to end the human immunodeficiency virus (HIV) epidemic by 2030 [1, 2], the Joint United Nations Programme on HIV/AIDS (UNAIDS), in 2014, set ambitious “90–90–90” targets for accelerating antiretroviral therapy (ART) coverage [1] in order to reduce HIV transmission [3–6] and HIV-related morbidity and mortality [6] worldwide. The UNAIDS 90–90–90 targets aim for 90% of people living with HIV to know their HIV status, 90% of people with diagnosed HIV infection to receive ART, and 90% of people receiving ART to achieve viral suppression [1, 2]. In 2018, the Sub-Saharan Africa region had an estimated 25.6 million people living with HIV (68% of all people living with HIV globally) and 1.08 million new HIV infections. By 2018, the region had achieved 64% ART coverage, with 16.4 million people accessing ART [7]. Increasing ART coverage in the Sub-Saharan Africa region is a global priority. Kenya has an adult HIV prevalence of 4.7%, and in 2018, had an estimated 1.6 million people living with HIV and 46,000 new HIV infections [8]. By September 2018, Kenya had achieved 71% ART coverage with over 1.14 million people accessing ART [9].

Identifying HIV-positive individuals through HIV testing services is the single most important step to increasing ART coverage. In line with the 2015 World Health Organization HIV testing guidelines for generalized HIV epidemics [10], the 2015 Kenya HIV testing guidelines recommend provider-initiated testing and counseling for all patients attending health facilities [11]. In the last decade, Sub-Saharan Africa countries, including Kenya, have made dramatic progress in increasing the coverage of HIV testing [12–16], leading to fewer undiagnosed people living with HIV. Consequently, the percent yield of HIV diagnoses from universal provider-initiated testing and counseling in outpatient settings has decreased over time. In Kenya, 6,103,757 outpatients were tested for HIV in 2019 accounting for 60% of all HIV tests conducted, and 64,493 (1.06%) had an HIV-positive result [9]. Evidence-based strategies to better target HIV testing could increase the HIV-positive yield and improve testing efficiency.

Screening algorithms for HIV testing based on clinical characteristics have been evaluated among children and adolescents [17–21]; however, their use is limited as current guidelines recommend early HIV diagnosis and immediate initiation of ART regardless of clinical status [6, 22–24]. Multiple studies in the United States have evaluated behavior-based risk-score algorithms to better target routine HIV testing [25–27]. Comparable studies in Sub-Saharan Africa are limited. One study conducted in Malawi evaluated the use of a risk-score algorithm to identify acute (pre-seroconversion) HIV infection among sexually transmitted infection (STI) clinic attendees [28]. Despite the paucity of studies evaluating risk-score algorithms for routine HIV testing in Sub-Saharan Africa, many studies conducted in this region have documented the association of certain behaviors with higher risk of HIV infection, including: polygamous marriage [29, 30], widowed [31–34] or separated/divorced status [32–34]; having a higher number of sexual partners [31, 35], sex in exchange for money or other favors [36, 37], or casual heterosexual sex [38]; being a sex worker, or man who has sex with men [38]; injection drug use [38]; fish trade [38]; inconsistent condom use [35]; use of alcohol before sex [39, 40]; intimate partner violence [41]; having an HIV infected sexual partner [42]; having an STI [31, 35]; and uncircumcised status in men [43–45].

To inform targeted HIV testing, we developed and validated a risk-score algorithm that incorporates sexual behavioral characteristics and assessed its performance among adults attending routine outpatient services at selected health facilities in Kenya.

## Methods

### Study Design

Using a retrospective study design, routinely collected HIV testing data from five health facilities in the western region of Kenya were used to develop a sociodemographic and behavioral characteristics-based risk-score algorithm for targeting HIV testing. Data from one high-volume facility were used to externally validate the algorithm. Development and validation of the risk-score algorithm followed systematic methodology that has been well described [46–49].

### Study Sites and Setting

Homa Bay, Siaya and Kisumu Counties have the highest HIV prevalence (range 16%–21%) in Kenya, and combined, have approximately 384,000 people living with HIV [50]. These counties accounted for 23% of all outpatients tested for HIV nationally in 2017, with 1,696,836 adults tested and 23,805 HIV-positive patients identified (1.4% HIV-positive yield) [51]. Data from seven health facilities that had the highest (1000–5000) average monthly outpatient department visits in the three counties were considered for inclusion in our analysis. Although all seven of the selected health facilities routinely collected HIV behavioral risk data as part of HIV testing and counseling services, one facility was found to inconsistently document behavioral risk information and was therefore excluded.

The six health facilities included in our study offered provider-initiated HIV testing and counseling to outpatients using an opt-out approach. This included screening for HIV-testing eligibility, and provision of pre-test counseling, testing, and post-test counseling to eligible clients. Eligibility for HIV testing was based on the 2015 Kenya Ministry of Health HIV testing guidelines [11], which recommend testing individuals who have never been tested for HIV; individuals whose last reported negative HIV test result was more than 12 months ago, or who do not know the date of their most recent HIV test; individuals who have signs, symptoms, or a diagnosis of tuberculosis or STI; and those who report recent HIV exposure. In March 2017, eligibility for HIV testing was expanded in order to increase access to HIV testing services. The expanded eligibility criteria included individuals reporting a negative HIV test result in the past 3 to 12 months, and those reporting a negative HIV test result in the past < 3 months, but for whom the test result could not be confirmed in clinic records. Eligible patients were tested for HIV according to the Ministry of Health guidelines using Determine™ and First Response™ rapid point-of-care kits; an individual was considered HIV-negative (uninfected) if the Determine test result was negative, HIV-positive (infected) if the Determine and First Response serial test results were positive, and inconclusive if the Determine result was positive and the First Response result was negative. From September 2017, the health facilities in our study used standardized forms to document behavioral risk characteristics routinely assessed by HIV-testing counselors to guide HIV prevention counseling during pre-test counseling sessions.

Our analysis included data from clients aged 15 years and older who were tested for HIV between September 2017 and May 2018 in the outpatient departments of the 6 study sites, and who had documentation of one or more behavioral risk characteristics. Records for patients with inconclusive HIV test results were excluded. At the six health facilities, data for an entire month were excluded if ≥ 50% of patients tested for HIV in that month did not have any documentation of behavioral risk characteristics.

### Data Management

Sociodemographic, HIV screening and testing, and behavioral risk information were recorded manually on Ministry of Health registers and standardized forms. At each health facility, the data were reviewed for completeness and accuracy, and entered into a secure password-protected database with in-built data consistency checks. Data meeting the study inclusion criteria were stripped of all identifiers (names and unique patient numbers), assigned new evaluation-specific identification numbers, entered into a study-specific secure password-protected database, and encrypted. Encrypted de-identified data were uploaded from each facility to a central database.

### Risk-Score Algorithm Development

Adult outpatient HIV testing data from five of the six health facilities included in our study were used to develop overall and gender-specific risk-score algorithms; two facilities were in Kisumu County (a referral hospital and sub-county hospital), two were in Homa Bay County (a county and sub-county hospital), and one was in Siaya County (a county hospital). These five facilities accounted for approximately 7% of adult outpatients tested for HIV in the three counties in 2017.

The primary outcome in this analysis was an HIV-positive test result. Sociodemographic and behavioral characteristics were considered for inclusion in the development of the predictive model if they were among those routinely collected during the pre-test counseling phase of HIV testing, and have been shown [27, 33, 52–54] or hypothesized to be associated with HIV infection. These included: sociodemographic characteristics (sex, age, marital status and occupation); behavioral characteristics (change in sexual partners, number of sexual partners, consistent condom use, had sex in exchange for money/favors, engaged in sex work, men who reported having sex with men, female anal sex, injecting drugs for pleasure, had sex under the influence of alcohol or other substance, and coerced to have sex); reported treatment for STI; circumcision status; and specific reasons for HIV testing eligibility (never tested for HIV, interval since last HIV-negative test, having tuberculosis, having an STI, and reporting recent HIV exposure). Characteristics such as education level, having an HIV infected sexual partner [52] and involvement in fish trade [38], which have been shown to be associated with HIV infection in other studies, were not routinely collected.

Development of the HIV infection predictive model was conducted in a systematic fashion, using univariable and multivariable analyses. As recommended for continuous variables [55–57], the association between age and HIV infection were assessed using a generalized additive model; the predicted odds of HIV-positivity by age were plotted, and informed age categorization into 5-year bands. The age-bands were further categorized into groups according to their HIV prevalence (the proportion of HIV infected individuals) as follows: ages 15–19, 20–24 and ≥ 50 years (HIV prevalence range of 0.33%–0.99%); ages 25–29, 30–34 and 45–49 years (HIV prevalence range of 1.32%–1.68%); and ages 35–39 and 40–44 years (HIV prevalence range of 1.97%–2.49%).

Univariable analysis was conducted to assess the independent association between the sociodemographic and behavioral characteristics and HIV infection, by computing odds ratios (ORs) and their corresponding 95% confidence intervals (CIs) and p values (significant at p ≤ 0.05). Two variables were not included in the univariable analysis: having sex in the prior 12 months, as multiple characteristics were assessed only for those who had sex in the prior 12 months, and consistent condom use with a sexual partner, as the documentation format made this variable difficult to interpret.

The initial full multivariable analysis included all variables with a significant higher odds (OR > 1.0) of HIV infection in univariable analysis, and those selected based on prior knowledge of an association with HIV infection. The variables in the full multivariable analysis were evaluated in a stepwise multivariable logistic regression, that incorporated Akaike information criterion for model selection, to identify the model/algorithm that best predicted HIV infection. Corresponding ORs, β regression coefficients and 95% CIs were computed. All participants with missing data were excluded from the univariable and multivariable analyses.

The final model was internally validated using 10-fold cross-validation. The ability of the final risk-score algorithm to discriminate between individuals with, and without, HIV infection was evaluated by computing the average area under the receiver operating curve (AUC, the area under a plot of sensitivity and the inverse of specificity) from the ten different cross-validation models. R-squared (R^2^) was computed to assess the extent to which the HIV prevalence variability can be explained by the model.

Risk-scores for each variable in the final model were created by multiplying the corresponding β regression coefficient by 10 and rounding to the nearest integer for ease of calculation. Each patient’s total risk-score was generated by summing the scores for all variables met.

To create risk-score categories, patient risk-scores were arranged in ascending order. The corresponding HIV prevalence for patients meeting each score was computed and used to identify mutually exclusive cut-points for unique risk-score groupings. The aggregate HIV prevalence and corresponding CIs were then calculated for each defined risk-score grouping.

### Risk-Score Algorithm Validation

Data from Kisumu County Hospital, a health facility among the six high-volume sites selected for inclusion in our study, were used to externally validate the overall and the gender-specific risk-score algorithms developed. This hospital had the highest number (~ 38,000) of adult outpatients tested for HIV in 2017 in the three Counties of Siaya, Kisumu and Homa Bay. Procedures for HIV testing, documentation of sociodemographic and behavioral characteristics, and management of HIV testing data were similar to those earlier described for the other facilities included in the study.

For validation, each patient’s risk-score was generated using the risk-score algorithm developed, and patients were grouped into respective risk-score categories. HIV prevalence and corresponding CIs for each risk-score category were then calculated. The AUC and R^2^ were computed in order to assess the algorithm’s discrimination performance, and the extent to which variability in HIV prevalence is explained by the model, respectively.

### Data Analysis

Data were managed using Stata Statistical Software version 14 (StataCorp, College Station, TX) and R version 3.6.2 [58]. The Classification And REgression Training (caret) package for predictive modelling was used to perform 10-fold cross-validation and to compute the AUC and R^2^.

### Ethical Considerations

The Institutional Review Board of Kenyatta National Hospital (Nairobi, Kenya) approved the protocol to conduct this analysis. The protocol was also reviewed and approved according to the human research protection procedures for the United States Centers for Disease Control and Prevention (Atlanta, Georgia).

## Results

### Characteristics of Patients at the Five Health Facilities Used for Risk-Score Algorithm Development

Out of the 45 total months (9 months for each of the 5 health facilities) that data were eligible for inclusion in the study, data for 37 (82%) months met the inclusion criteria. During these months, 99.9% (27,685/27,692) of adults attending OPD services were screened for HIV testing eligibility, and 87% (21,764/24,966) of those eligible were tested for HIV. Of 21,745 patients with positive or negative HIV test results, 19,458 (89%) had behavioral risk characteristics documented and were included in our analysis.

Among the 19,458 patient records included, the median age was 29 years (interquartile range 22–43 years) and 11,149 (57%) were women (Table 1). Most patients [10,731 (61%)] were in monogamous marriage, and approximately two-thirds were either in trade/sales/service occupation [5467 (29%)] or were school/college going [5167 (27%)]. The majority of patients [18,450 (95%)] reported having sex in the prior 12 months, of whom 5038 (28%) reported having 2 or more sexual partners, and 2749 (17%) reported changes in sexual partners. Among those with changes in sexual partners, 1411 (51%) reported new sexual partners and 800 (29%) were widowed. Few patients reported having sex in exchange for money/favors/gifts [773 (4%)], having sex under the influence of alcohol/other substances [496 (3%)], having been coerced to have sex [480 (3%)], or having received treatment for STI in the prior 12 months [251 (1%)]. A minority of patients had never been tested for HIV [688 (3%)] or had a negative HIV test result > 12 months prior [12 (0.1%)] (Table 1). Overall, 210 (1.1%) patients were HIV-positive.

**Table 1:** Characteristics of outpatient attendees by gender at five high-volume facilities (Jaramogi Oginga Odinga Teaching and Referral Hospital, Homa Bay County Hospital, Siaya County Hospital, Ahero Sub-county Hospital, Mbita Sub-county Hospital) used for algorithm development

Characteristic	All patients	Men	Women	p value
	n (%)	n (%)	n (%)	
Total	19,458	8309	11,149	
Sociodemographic characteristics				
Age in years, median (interquartile range)	29 (22–43)	30 (22–43)	29 (22–45)	
Age categories				
15–19, 20–24 and ≥ 50 years	10,577 (54)	4399 (53)	6178 (55)	0.042
25–29, 30–34 and 45–49 years	6211 (32)	2668 (32)	3543 (32)	1.00
35–39 and 40–44 years	2670 (14)	1242 (15)	1428 (13)	0.14
Marital status				
Never married	4546 (26)	2248 (30)	2298 (23)	< 0.001
Married monogamous	10,731 (61)	4312 (57)	6419 (64)	< 0.001
Married polygamous	952 (6)	667 (9)	285 (3)	< 0.001
Cohabiting	239 (1)	59 (1)	180 (2)	0.61
Separated/divorced	224 (1)	118 (1)	106 (1)	0.54
Widowed	948 (5)	181 (2)	767 (7)	0.004
Occupation				
Professional/administrative/clerical	2180 (11)	1011 (12)	1169 (11)	0.15
Manual^a^/domestic	1044 (6)	931 (12)	113 (1)	< 0.001
Agriculture	2285 (12)	1050 (13)	1235 (11)	0.14
Trade/sales/service	5467 (29)	1926 (24)	3541 (32)	< 0.001
Unemployed	2924 (15)	863 (11)	2061 (19)	< 0.001
School/college going	5167 (27)	2282 (28)	2885 (26)	0.11
Behavioral characteristics				
Had sex in the prior 12 months				
Yes	18,450 (95)	7840 (94)	10,610 (95)	0.003
No	1008 (5)	469 (6)	539 (5)	0.49
Number of sexual partners in the prior 12 months				
1	13,220 (72)	5084 (65)	8136 (78)	< 0.001
≥2	5038 (28)	2681 (35)	2357 (22)	< 0.001
Changes in sexual partners in the prior 12 months				
Not had change in sexual partner	13,523 (83)	5459 (81)	8064 (85)	< 0.001
New sexual partner	1411 (8)	867 (13)	544 (6)	< 0.001
Newly married	155 (1)	69 (1)	86 (1)	1.00
Ended a sexual relationship	293 (2)	131 (2)	162 (2)	1.00
Divorced/separated	90 (1)	44 (1)	46 (0.4)	0.49
Widowed	800 (5)	175 (2)	625 (6)	0.051
Had sex in exchange for money/favors in the prior 12 months^b^	n = 17,373	n = 7358	n = 10,015	
Yes	773 (4)	305 (4)	468 (5)	0.52
Had sex under influence of alcohol/other substance in the prior 12 months	n = 17,366	n = 7354	n = 10,012	
Yes	496 (3)	321 (4)	175 (2)	0.23
Coerced to have sex in the prior 12 months	n = 17,094	n = 7274	n = 9820	
Yes	480 (3)	97 (1)	383 (4)	0.15
Reported treatment for STI in the prior 12 months	n = 16,928	n = 7188	n = 9740	
Yes	251 (1)	121 (2)	130 (1)	0.51
Engaged in sex work, men who have with men, female anal sex, injecting drugs for pleasure in the prior 12 months^b^	n = 16,450	n = 6960	n = 9490	
Yes	730 (4)	294 (4)	436 (5)	0.53
Circumcision status (men only)		n = 6158		
Circumcised		4871 (78)		
HIV testing information				
Reason for HIV testing eligibility				
Never been tested for HIV	688 (3)	358 (4)	330 (3)	0.48
HIV negative test > 12 months prior	12 (0.1)	7 (0.08)	5 (0.05)	0.84
HIV negative test 6 to 12 months prior	5967 (31)	2651 (32)	3316 (30)	0.10
HIV negative test 3 to 6 months prior	9454 (49)	3913 (47)	5541 (50)	0.004
HIV negative test < 3 months ago (unverified)	2666 (14)	1067 (13)	1599 (14)	0.46
HIV negative test date unknown	668 (3)	311 (4)	357 (3)	0.48
Has tuberculosis, STI or recent HIV exposure	3 (0.02)	2 (0.02)	1 (0.009)	0.95

*n* number, *STI* sexually transmitted infection

^a^Manual occupation refers to both skilled and unskilled

^b^Due to multicollinearity, the characteristic “engaged in sex work, men who have with men, female anal sex, injecting drugs for pleasure in the prior 12 months” was excluded in univariable and multivariable analysis, while the characteristic “had sex in exchange for money/favors in the prior 12 months” was included

Compared to women, a significantly higher proportion of men were never married (30% vs 23%, p < 0.001), in a polygamous marriage (9% vs 3%, p < 0.001), in a manual/domestic occupation (12% vs 1%, p < 0.001), had ≥ 2 sexual partners (35% vs 22%, p < 0.001) and reported a new sexual partner in the prior 12 months (13% vs 6%, p < 0.001). Conversely, a significantly higher proportion of women were in monogamous marriage (64% vs 57%, p < 0.001), widowed (7% vs 2%, p 0.004), in a trade/sale/service occupation (32% vs 24%, p < 0.001), unemployed (19% vs 11%, p < 0.001), or reported being widowed in the prior 12 months (6% vs 2%, p 0.051, Table 1).

### Overall Risk-Score Algorithm Development

The following characteristics were positively significantly associated with HIV infection in univariable analysis: being aged 35–39 and 40–44 years; male gender; manual/domestic and trade/sales/service occupation; polygamous marriage, separated/divorced or widowed; in the prior 12 months having a new sexual partner, ≥ 2 sexual partners, or reporting treatment for STI; having never been tested for HIV; or having a negative HIV test result > 12 months prior (Table 2).

**Table 2:** Univariable association of sociodemographic and behavioral characteristics with HIV infection at five high-volume facilities used for algorithm development

Characteristic	Number HIV positive/tested (%)	Univariable analysis^a^	p value
		Odds ratio (95% CI)	
Total	210/19,458 (1.08)		
Ages 15–19, 20–24 and ≥ 50 years	74/10,577 (0.70)	0.46 (0.32, 0.64)	< 0.001
Ages 25–29, 30–34 and 45–49 years	79/6211 (1.27)	1.2 (0.87, 1.66)	0.28
Ages 35–39 and 40–44 years	57/2670 (2.13)	2.39 (1.68, 3.39)	< 0.001
Men	110/8309 (1.32)	1.46 (1.06, 2.01)	0.019
Women	100/11,149 (0.90)	0.68 (0.5, 0.94)	0.019
Never married	32/4546 (0.70)	0.5 (0.31, 0.83)	0.006
Married monogamous	112/10,731 (1.04)	0.74 (0.53, 1.02)	0.06
Married polygamous	20/952 (2.10)	2.11 (1.25, 3.56)	0.005
Cohabiting	2/239 (0.84)	0.88 (0.22, 3.58)	0.86
Separated/divorced	16/224 (7.14)	7.56 (4.01, 14.25)	< 0.001
Widowed	17/948 (1.79)	2.3 (1.32, 4.02)	0.003
Professional/administrative/clerical occupation	22/2180 (1.01)	0.71 (0.42, 1.22)	0.22
Manual/domestic occupation	22/1044 (2.11)	2.06 (1.26, 3.39)	0.004
Trade/sales/service occupation	93/5467 (1.70)	1.84 (1.33, 2.53)	< 0.001
Agriculture occupation	22/2285 (0.96)	0.82 (0.49, 1.39)	0.47
School/college going	18/5167 (0.35)	0.24 (0.13, 0.45)	< 0.001
Unemployed	28/2924 (0.96)	1.22 (0.77, 1.95)	0.39
≥ 2 Sexual partners in the prior 12 months	83/5038 (1.65)	2.29 (1.62, 3.22)	< 0.001
No change in sexual partners in prior 12 months	126/13,523 (0.93)	0.52 (0.36, 0.77)	0.001
New sexual partner in prior 12 months	27/1411 (1.91)	2.61 (1.69, 4.03)	< 0.001
Newly married in prior 12 months	1/155 (0.65)	0.66 (0.09, 4.78)	0.68
Ended a sexual relationship in prior 12 months	2/293 (0.68)	0 (0, 1.01)	0.97
Divorced/separated in prior 12 months	4/90 (4.44)	3.09 (0.75, 12.76)	0.12
Widowed in prior 12 months	6/800 (0.75)	1.03 (0.45, 2.34)	0.94
Had sex in exchange for money/favors in prior 12 months	13/773 (1.68)	1.58 (0.83, 3.02)	0.16
Had sex under influence of alcohol/other substance in prior 12 months	5/496 (1.01)	0.77 (0.24, 2.42)	0.65
Coerced to have sex in prior 12 months	8/480 (1.67)	1.43 (0.63, 3.26)	0.39
Reported treatment for STI in prior 12 months	9/251 (3.59)	3.34 (1.55, 7.22)	0.002
Never been tested for HIV	32/688 (4.65)	5.44 (3.4, 8.71)	< 0.001
HIV negative test > 12 months prior	1/12 (8.33)	13.89 (1.7, 113.55)	0.014
HIV negative test 6 to 12 months prior	63/5967 (1.06)	1.13 (0.8, 1.58)	0.49
HIV negative test 3 to 6 months prior	74/9454 (0.78)	0.61 (0.44, 0.85)	0.003
HIV negative test < 3 months ago (unverified)	36/2666 (1.35)	0.93 (0.57, 1.53)	0.79
HIV negative test date unknown	4/668 (0.60)	0.58 (0.19, 1.84)	0.36
Has tuberculosis, STI or recent HIV exposure	0/3 (0.00)		

*n* number, *CI* confidence interval, *STI* sexually transmitted infection

^a^Missing data omitted from univariable analysis

The initial full multivariable analysis included all the variables that were positively significantly associated with HIV infection in the univariable analysis. Additional variables that were also included based on known association with HIV infection were: divorced/separated or widowed, in the prior 12 months having sex in exchange for money/favors and coerced to have sex (Table 3). The AUC for the full model was 0.66 (95% CI 0.44–0.88).

**Table 3:** Multivariable association of sociodemographic and behavioral characteristics with HIV infection among outpatient attendees at five high-volume facilities

	Full multivariable model		Stepwise multivariable analysis		
	Odds ratio	β (95% CI)	Odds ratio	β (95% CI)	Risk score^a^
Ages 35–39 and 40–44 years	2.12	0.75 (0.38, 1.12)	2.16	0.77 (0.4, 1.14)	8
Men	1.16	0.15 (− 0.21, 0.52)			
Manual/domestic occupation	1.99	0.69 (0.11, 1.26)	2.20	0.79 (0.23, 1.35)	8
Trade/sales/service occupation	1.92	0.65 (0.29, 1)	1.95	0.67 (0.31, 1.02)	7
Married polygamous	1.55	0.44 (− 0.15, 1.04)	1.80	0.59 (0.01, 1.17)	6
Widowed	3.90	1.36 (0.66, 2.06)	2.39	0.87 (0.26, 1.48)	9
Separated/divorced	6.96	1.94 (1.19, 2.68)	5.26	1.66 (0.98, 2.34)	17
≥ 2 Sexual partners in prior 12 months	1.63	0.49 (− 0.04, 1.02)	1.58	0.46 (0.06, 0.86)	5
New sexual partner in prior 12 months	1.27	0.24 (− 0.4, 0.88)			
Divorced/separated in prior 12 months	0.33	− 1.1 (− 2.73, 0.52)			
Widowed in prior 12 months	0.39	− 0.95 (− 2.03, 0.13)			
Coerced to have sex in prior 12 months	1.32	0.28 (− 0.61, 1.17)			
Had sex in exchange for money/favors in prior 12 months	0.90	− 0.11 (− 0.85, 0.63)			
Reported treatment for STI in prior 12 months	2.61	0.96 (0.13, 1.79)	2.97	1.09 (0.29, 1.9)	11
Never been tested for HIV	6.23	1.83 (1.34, 2.33)	6.17	1.82 (1.33, 2.31)	18
HIV negative result > 12 months ago	7.92	2.07 (− 0.14, 4.29)	9.03	2.2 (0.02, 4.37)	22

*β* Regression coefficient, *CI* confidence interval, *STI* sexually transmitted infection

^a^Computed by multiplying the β regression coefficients by 10 and rounding to the nearest integer

The final best-fit model/risk-score algorithm consisted of the following variables: age category 35–39/40–44 years; occupation (manual/domestic or trade/sales/service); marital status (polygamous marriage, separated/divorced or widowed); in the prior 12 months having ≥ 2 sexual partners or reporting treatment for an STI; and having never been tested for HIV or having a negative HIV test result > 12 months prior (Table 3). The final model/algorithm had an AUC of 0.69 (95% CI 0.53–0.84) and R^2^ of 0.89.

The variables in the final algorithm were each assigned a risk-score, and each patient’s risk-score was calculated as the sum of risk-scores for variables met. Patients were grouped into the following 4 risk-score categories: ≤ 9 [HIV prevalence 0.6% (95% CI 0.46–0.75)], 10–15 [HIV prevalence 1.35% (95% CI 0.85–1.84)], 16–29 [HIV prevalence 2.65% (95% CI 1.8–3.51)], and ≥ 30 [HIV prevalence 15.15% (95% CI 9.03–21.27)] (Table 4). The 3 highest risk-score categories (score ≥ 10) accounted for 55% of HIV-positive patients identified, yet represented just 24% of the total patients tested for HIV. Similarly, patients in the 2 highest risk-score categories (score ≥ 16) accounted for 37% of HIV-positive patients identified, yet represented just 10% of the total patients tested for HIV.

**Table 4:** Final algorithm risk-score categories for development and validation datasets

Risk-score category	Number HIV positive/tested	HIV prevalence, % (95% CI)	% of total HIV positive	% of total tests
Risk-score categories for algorithm development dataset				
≤ 9	68/11,289	0.6 (0.46, 0.75)	45	76
10–15	28/2076	1.35 (0.85, 1.84)	18	14
16–29	36/1357	2.65 (1.8, 3.51)	24	9
≥ 30	20/132	15.15 (9.03, 21.27)	13	1
Total^a^	152/14,854	1.02%		
Risk-score categories for algorithm validation dataset				
≤ 9	79/8142	0.97 (0.76, 1.18)	51	77
10–15	28/1207	2.32 (1.47, 3.17)	18	11
16–29	44/1193	3.69 (2.62, 4.76)	28	11
≥ 30	5/74	6.76 (1.04, 12.48)	3	1
Total^a^	156/10,616	1.47%		

*CI* confidence interval

^a^Patients with missing data omitted from the analysis

### Overall Risk-Score Algorithm Validation

The validation dataset consisted of 11,330 patient records, of which 174 (1.6%) were HIV-positive. The sociodemographic and behavioral characteristics of patients in the validation dataset are shown in Table 5. In comparison to the development dataset, the validation dataset had a significantly higher proportion of patients with manual/domestic and trade/sales/service occupation, and a significantly lower proportion of patients who reported having ≥ 2 sexual partners in the prior 12 months, and having a negative HIV test result > 12 months prior (Table 5).

**Table 5:** Comparison of characteristics of patients at the five health facilities used for algorithm development and one facility used for algorithm validation

Characteristic	Five health facilities^a^	One health facility^b^	p value
	n (%)	n (%)	
Total	19,458	11,330	
Sociodemographic characteristics			
Age in years, median (interquartile range)	29 (22–43)	27 (22–38)	
Age categories			
15–19, 20–24 and ≥ 50 years	10,577 (54)	5744 (51)	< 0.001
25–29, 30–34 and 45–49 years	6211 (32)	4089 (36)	< 0.001
35–39 and 40–44 years	2670 (14)	1497 (13)	0.37
Gender			
Men	8309 (43)	4706 (42)	0.27
Women	11,149 (57)	6624 (58)	0.19
Marital status			
Never married	4546 (26)	3968 (35)	< 0.001
Married monogamous	10,731 (61)	6191 (55)	< 0.001
Married polygamous	952 (6)	465 (4)	0.4
Cohabiting	239 (1)	11 (0.1)	0.74
Separated/divorced	224 (1)	154 (1)	1
Widowed	948 (5)	532 (5)	1
Occupation			
Professional/administrative/clerical	2180 (11)	1086 (10)	0.38
Manual/domestic	1044 (6)	1000 (9)	< 0.001
Agriculture	2285 (12)	643 (6)	< 0.001
Trade/sales/service	5467 (29)	3783 (34)	< 0.001
Unemployed	2924 (15)	1974 (18)	0.005
School/college going	5167 (27)	2531 (23)	< 0.001
≥ 2 Sexual partners in prior 12 months	5038 (28)	1181 (11)	< 0.001
Reported treatment for STI in prior 12 months	251 (1)	74 (1)	1
Reason for HIV testing eligibility			
Never been tested for HIV	688 (4)	695 (6)	0.08
HIV negative test > 12 months prior	12 (0.1)	2 (0.02)	< 0.001
HIV negative test 6 to 12 months prior	5967 (31)	3623 (32)	0.31
HIV negative test 3 to 6 months prior	9454 (49)	4836 (43)	< 0.001
HIV negative test < 3 months ago (unverified)	2666 (14)	2074 (18)	< 0.001
HIV negative test date unknown	668 (3)	99 (1)	0.26
Has tuberculosis, STI or recent HIV exposure	3 (0.02)	1 (0.01)	< 0.001

*n* number, *STI* sexually transmitted infection

^a^Jaramogi Oginga Odinga Teaching and Referral Hospital, Homa Bay County Hospital, Siaya County Hospital, Ahero Sub-county Hospital, Mbita Sub-county Hospital

^b^Kisumu County Hospital

When applied to the validation dataset, the final risk-score algorithm/model had an AUC of 0.69 (95% CI 0.60–0.77) and R^2^ of 0.88. The risk score categories ≤ 9, 10–15, 16–29 and ≥ 30 had an increasing HIV prevalence of 0.97% (95% CI 0.76–1.18), 2.32% (95% CI 1.47–3.17), 3.69% (95% CI 2.62–4.76) and 6.76% (95% CI 1.04–12.48), respectively (Table 4). The 3 highest risk-score categories (score ≥ 10) accounted for 49% of HIV-positive patients identified, but only 23% of the total patients tested for HIV. The 2 highest risk-score categories (score ≥ 16) accounted for 31% of HIV-positive patients identified, but only 12% of the total patients tested for HIV.

### Development of Gender-Specific Risk-Score Algorithms

Characteristics that were positively significantly associated with HIV infection in univariable analysis (OR > 1.0 at p ≤ 0.05) among men and women are shown in Supplementary Table SI. Full multivariable models for men and women are shown in Supplementary Tables SII and SIII. The AUC for the full model was 0.75 (95% CI 0.65–0.85) among men and 0.68 (95% CI 0.56–0.8) among women.

The final best-fit model/risk-score algorithm among men had an AUC of 0.76 (95% CI 0.56–0.96) and an R^2^ of 0.69, and consisted of the following variables: age categories 25–29/30–34/45–49 years and 35–39/40–44 years; occupation (manual/domestic or trade/sales/service); marital status (separated/divorced or widowed); in the prior 12 months having ≥ 2 sexual partners or a new sexual partner; circumcised status; and having never been tested for HIV (Supplementary Table SII).

The final risk-score algorithm among women had an AUC of 0.66 (95% CI 0.47–0.85) and an R^2^ of 0.87, and consisted of the following variables: age category 35–39/40–44 years; trade/sales/service occupation; marital status (polygamous marriage, separated/divorced or widowed); in the prior 12 months having a new sexual partner or reporting treatment for an STI; and having never been tested for HIV or having a negative HIV test result > 12 months prior (Supplementary Table SIII).

Risk-score categories and corresponding HIV prevalence among men and women are shown in Supplementary Table SIV. Among men, the 3 highest risk-score categories (score ≥ 13) accounted for 86% of HIV-positive patients identified, yet represented 50% of the total patients tested for HIV. Similarly, among women, the 3 highest risk-score categories (score ≥ 8) accounted for 51% of HIV-positive patients identified, yet represented 23% of the total patients tested for HIV (Supplementary Table SIV).

### Validation of the Gender-Specific Risk-Score Algorithm

The validation dataset comprised 4706 (42%) men and 6624 (58%) women. When applied to the validation dataset, the final algorithm/model had an AUC of 0.71 (95% CI 0.57–0.86) and an R^2^ of 0.85 among men, and an AUC of 0.66 (95% CI 0.49–0.84) and an R^2^ of 0.95 among women. The risk-score categories and corresponding HIV prevalence among men and women are shown in Supplementary Table SIV.

## Discussion

Our study demonstrates that a HIV predictive risk-score algorithm, derived from a set of sociodemographic and behavioral characteristics, can be used to identify sub-populations who have higher risk of HIV infection to whom HIV testing could be targeted. Other studies, which have evaluated similar risk-score algorithms for targeting HIV testing, have been conducted in specific settings (STI clinics, a methadone clinic and a blood donor center) in the United States [53, 59]. Although the Denver risk-score algorithm (also evaluated in the United States) has been widely validated, including in general outpatient care settings [25, 26, 60], it was developed using data from STI clinic attendees [27, 61]. To our knowledge, our study is the first to develop and validate an HIV testing algorithm using data from the general outpatient care setting, and the first of its kind to be conducted in the Sub-Saharan Africa setting.

Our risk-score algorithm consists of simple variables, which in our study were collected within a routine health care delivery setting, demonstrating the feasibility of implementation. The overall final algorithm comprised the following variables: age category 35–39/40–44 years; occupation (manual/domestic or trade/sales/service); marital status (polygamous marriage, separated/divorced or widowed); in the prior 12 months having ≥ 2 sexual partners or reporting treatment for an STI; and having never been tested for HIV or having a negative HIV test result > 12 months prior. This algorithm accounted for a high proportion of the variability of HIV prevalence in our development (R^2^ 0.89) and validation (R^2^ 0.88) study populations. The algorithm’s ability to discriminate between individuals with, and without, HIV infection in the general outpatient setting was modest (AUC of 0.69 for both the development and validation datasets) and comparably lower than the Denver HIV risk-score algorithm (AUC range of 0.75–0.85) [25, 27, 60]. This likely reflects more widespread distribution of HIV-risk factors among persons accessing health facilities in the setting of a generalized HIV epidemic, although ways to improve the discrimination performance of the overall algorithm should be explored.

Among women, the proportion of variability in HIV prevalence accounted for by the final model/algorithm was high (R^2^ of 0.87 in the development and 0.95 in the validation datasets), and varied among men (R^2^ of 0.69 and 0.85 in the development and validation datasets, respectively). Performance of the algorithm in discriminating patients with, and without, HIV infection was modest among women (AUC of 0.66 for both the development and validation datasets), and somewhat higher among men (AUC of 0.76 and 0.71 for the development and validation datasets, respectively). Although our study highlights variation in the performance of gender-specific algorithms, majority of the HIV-risk factors included in the final models were similar for both sexes. Use of a single overall algorithm may, therefore, be appropriate and likely more feasible to implement in the field.

The risk-score algorithm presented offers an evidence-base to guide identification of outpatient sub-populations with higher risk for HIV, to whom HIV testing could be prioritized. Our study found that targeted HIV testing using the three highest risk-score categories in the overall algorithm, would dramatically reduce (by about 75%) the number of patients tested; however, this approach would miss the diagnosis of approximately 50% of HIV infected individuals accessing health facilities, making the use of the algorithm inferior to universal testing. Even for the gender-specific algorithm among men, which had superior discrimination performance as compared to the overall algorithm, targeted HIV testing using the three highest risk-score categories would reduce the number of patients tested by one half, and miss the diagnosis of approximately 14% of HIV infected individuals. The algorithm’s use should, therefore, be considered in settings where resource or other logistical constraints necessitate targeted testing, and should be coupled with other HIV testing strategies recommended by the World Health Organization [10, 62].

The predictors included in our risk-score algorithm are consistent with those shown in other studies to be associated with higher risk of HIV infection. The pattern of HIV prevalence by age and sex is consistent with national surveys in Kenya [63]. Furthermore, several studies have shown that polygamous marriage [29, 30], widowed status [31–33], or separated/divorced status [32–34]; having multiple sexual partners [31, 34, 35]; having a new sexual partner [31, 35]; having an STI [31, 35]; and uncircumcised status among men [43–45] are associated with higher risk of HIV infection. Some studies have shown an association between HIV risk and higher socioeconomic status/employment/having income [31, 64–67], others have shown an association with low socioeconomic status [68, 69], while others have demonstrated a mixed association [70, 71] or no association [72–75]. Although we did not assess socioeconomic status directly, we found manual/domestic or trade/sales/service occupations were associated with higher risk of HIV infection, which might be explained by an unidentified interplay between source of income and behavior, including increased opportunity for social interaction and travel. Our findings are also consistent with program data from western Kenya which found that patients who had never been tested for HIV, or had a negative HIV test result > 12 months prior were more likely HIV-positive [76]. Most patients (95%) had been tested for HIV within the previous 12 months, reflecting intensified HIV testing efforts to increase ART coverage in the study region [77–80]. Although studies have shown that alcohol use [39, 40], intimate partner violence [41], and having sex in exchange for money/favors [81] are associated with higher risk of HIV infection, these were not significant in our study; possibly owing to these variables being under-reported or being less prevalent in our study population of general outpatient attendees. Although other studies have demonstrated an association of race/ethnicity with HIV infection [52], this association has not been shown by studies conducted in Kenya and was not evaluated in our study.

Behavioral risk data were collected by trained counselors at a private space, to facilitate patient privacy and reduce social desirability bias. However, comparison of our study’s patient characteristics with results from the most recent (2014) Kenya Demographic and Health Survey suggests patients might have under-reported certain variables. The survey reported that 1.7% of women and 22% of men in the study region use alcohol [82], suggesting that the proportion of patients in our study who reported having sex under the influence of alcohol (2%) is likely an underestimate. Similarly, whereas the survey results showed that nationally 7.8% of women and 2.3% of men experience sexual violence [82], our study found that 2% of patients reported being coerced to have sex in the prior 12 months, also likely an underestimate. The proportion of patients who reported having sex in exchange for money/favors (3%) in our study, is however, comparable to the national survey findings [82].

Our study had several limitations. First, our algorithm did not include all potential predictor variables, as education level, condom use and having an HIV-positive sexual partner were not included; however, we believe that the majority of behaviors that have been demonstrated to be associated with higher HIV infection in our study setting were included. Secondly, our study did not meet the sample size rule of ten outcome events per variable recommended for clinical predictive model evaluation [46, 83, 84]. Furthermore, by stratifying our analysis by gender, the sample size reduced further. However, studies that have evaluated the effect of the sample size recommendation have shown conflicting results [85–87], and further evaluation of the rule has been recommended [87, 88]. To minimize overfitting occasioned by a small sample size, our study incorporated the use of Akaike information criterion for variable selection in the step-wise regression model [57, 89]. Finally, although the development of our algorithm derives strength from using data from five health facilities located across three counties, data used for external validation was from a facility located in the same region. The algorithm should therefore be externally validated in other regions and settings, and the impact of its use evaluated.

## Conclusions

In summary, our study demonstrates that a HIV predictive risk-score algorithm, derived from a set of sociodemographic and behavioral characteristics, can be used to identify sub-populations who have higher risk of HIV infection to whom HIV testing could be targeted. The overall algorithm’s ability to discriminate between individuals with, and without, HIV infection in the general outpatient setting was modest. Additionally, using the three highest risk-score categories in the overall algorithm to target HIV testing would dramatically reduce (by about 75%) the number of patients tested, but miss the diagnosis of approximately 50% of HIV infected individuals accessing health facilities, making the use of the algorithm inferior to universal testing. Therefore, in settings where universal testing is not feasible, the risk-score algorithm offers an evidence-base to guide identification of patient sub-populations with higher HIV risk, to whom HIV testing could be targeted. Further evaluation is needed to explore ways to improve the discrimination performance of the algorithm, to externally validate the algorithm in other regions and settings, and to assess the impact of its use.

## Electronic supplementary material

Below is the link to the electronic supplementary material.

Supplementary file1 (DOCX 80 kb)

Supplementary file2 (ZIP 2030 kb)
