# Supplementary material for: Development and Validation of a Sociodemographic and Behavioral Characteristics-Based Risk-Score Algorithm for Targeting HIV Testing Among Adults in Kenya
Source: AIDS Behav. 2020 Jul 10;25(2):297–310. doi: 10.1007/s10461-020-02962-7 (PMC7846530; doi:10.1007/s10461-020-02962-7)
Supplement: Supplementary file 1 — Supplementary file1 (DOCX 80 kb) [file 10461_2020_2962_MOESM1_ESM.docx]

| **Supplementary Table SI: Univariable association of sociodemographic and behavioral characteristics with HIV infection by gender, at five high-volume facilities used for algorithm development** | | | | | | |
| --- | --- | --- | --- | --- | --- | --- |
| **Characteristic** | **Men** | | | **Women** | | |
|  | **Number HIV positive/ Tested** | **Univariable analysis^a^** | | **Number HIV positive/ Tested** | **Univariable analysis^a^** | |
|  |  | **Odds ratio (95% CI)** | **p value** |  | **Odds ratio (95% CI)** | **p value** |
| Total | 110/8,309 |  |  | 100/11,149 |  |  |
| Ages 15–19, 20–24 and >50 years | 36/4,399 | 0.48 (0.35, 0.67) | <0.001 | 38/6,178 | 0.41 (0.31, 0.54) | <0.001 |
| Ages 25–29, 30–34 and 45–49 years | 41/2,668 | 1.63 (1.19, 2.24) | 0.002 | 38/3,543 | 1.25 (0.95, 1.63) | 0.11 |
| Ages 35–39 and 40–44 years | 33/1,242 | 1.58 (1.07, 2.34) | 0.022 | 24/1,428 | 2.75 (2.06, 3.67) | <0.001 |
| Professional/administrative/clerical occupation | 7/1,011 | 0.63 (0.34, 1.17) | 0.15 | 15/1,169 | 0.93 (0.6, 1.45) | 0.75 |
| Manual/domestic occupation | 21/931 | 2.02 (1.26, 3.25) | 0.004 | 1/113 | 2.48 (1.7, 3.63) | <0.001 |
| Trade/sales/service occupation | 48/1,926 | 2.28 (1.66, 3.13) | <0.001 | 45/3,541 | 1.9 (1.45, 2.49) | <0.001 |
| Agriculture occupation | 15/1,050 | 0.65 (0.34, 1.23) | 0.18 | 7/1,235 | 0.84 (0.52, 1.37) | 0.49 |
| School/college going | 6/2,282 | 0.19 (0.1, 0.37) | <0.001 | 12/2,885 | 0.25 (0.15, 0.42) | <0.001 |
| Unemployed | 10/863 | 1.09 (0.72, 1.66) | 0.68 | 18/2,061 | 0.74 (0.49, 1.11) | 0.14 |
| Never married | 11/2,248 | 0.41 (0.27, 0.65) | <0.001 | 21/2,298 | 0.55 (0.39, 0.78) | <0.001 |
| Married monogamous | 64/4,312 | 0.93 (0.68, 1.28) | 0.67 | 48/6,419 | 0.94 (0.72, 1.23) | 0.65 |
| Married polygamous | 12/667 | 2.1 (1.24, 3.55) | 0.005 | 8/285 | 1.87 (1.15, 3.04) | 0.012 |
| Widowed | 5/181 | 1.12 (0.55, 2.3) | 0.76 | 12/767 | 1.77 (1.11, 2.82) | 0.016 |
| Separated/divorced^b^ | 11/118 | 9.29 (5.74, 15.04) | <0.001 | 5/106 | 6.46 (3.53, 11.82) | <0.001 |
| Cohabiting^c^ | 1/59 |  |  | 1/180 | 1.31 (0.32, 5.32) | 0.71 |
| >2 sexual partners in prior 12 months | 55/2,681 | 1.93 (1.38, 2.68) | <0.001 | 28/2,357 | 1.65 (1.23, 2.22) | <0.001 |
| No change in sexual partner in prior 12 months | 59/5,459 | 0.48 (0.32, 0.72) | <0.001 | 67/8,064 | 0.75 (0.51, 1.12) | 0.16 |
| New sexual partner in prior 12 months | 20/867 | 2.52 (1.57, 4.05) | <0.001 | 7/544 | 1.74 (1.08, 2.8) | 0.023 |
| Newly married in prior 12 months^cd^ | 1/69 |  |  | 0/86 |  |  |
| Ended a sexual relationship in prior 12 months^c^ | 0/131 |  |  | 2/162 | 0.4 (0.06, 2.9) | 0.37 |

| **Supplementary Table SI: continued….....** | | | | | | |
| --- | --- | --- | --- | --- | --- | --- |
| **Characteristic** | **Men** | | | **Women** | | |
|  | **Number HIV positive/ Tested** | **Univariable analysis^a^** | | **Number HIV positive/ Tested** | **Univariable analysis^a^** | |
|  |  | **Odds ratio (95% CI)** | **p value** |  | **Odds ratio (95% CI)** | **p value** |
| Divorced/separated in prior 12 months^b^ | 1/44 | 6.53 (2.59, 16.49) | <0.001 | 3/46 | 2.91 (0.71, 12.03) | 0.14 |
| Widowed in prior 12 months | 3/175 | 0.4 (0.1, 1.64) | 0.20 | 3/625 | 0.87 (0.39, 1.98) | 0.74 |
| Had sex in exchange for money/favors in prior 12 months | 9/305 | 1.58 (0.77, 3.23) | 0.22 | 4/468 | 1.05 (0.47, 2.39) | 0.90 |
| Had sex under influence of alcohol/other substance in prior 12 months | 3/321 | 1.09 (0.4, 2.97) | 0.86 | 2/175 | 1.91 (0.93, 3.9) | 0.08 |
| Coerced to have sex in prior 12 months^c^ | 0/97 |  |  | 8/383 | 1.54 (0.68, 3.49) | 0.30 |
| Reported treatment for STI in prior 12 months | 3/121 | 1.67 (0.52, 5.29) | 0.39 | 6/130 | 4.02 (2.03, 7.95) | <0.001 |
| Circumcised (men only) | 46/4,871 | 2.64 (1.59, 4.39) | <0.001 |  |  |  |
| Never been tested for HIV | 19/358 | 2.24 (1.31, 3.83) | 0.003 | 13/330 | 3.78 (2.59, 5.51) | <0.001 |
| HIV negative test >12 months prior^c^ | 0/7 |  |  | 1/5 | 16.12 (1.88, 138.55) | 0.011 |
| HIV negative test 6 to 12 months prior | 31/2,651 | 1.04 (0.74, 1.45) | 0.83 | 32/3,316 | 0.81 (0.6, 1.08) | 0.15 |
| HIV negative test 3 to 6 months prior | 39/3,913 | 0.69 (0.5, 0.96) | 0.025 | 35/5,541 | 0.7 (0.53, 0.92) | 0.010 |
| HIV negative test <3 months ago (unverified) | 18/1,067 | 1.28 (0.85, 1.9) | 0.23 | 18/1,599 | 1.16 (0.82, 1.64) | 0.41 |
| HIV negative test date unknown | 3/311 | 0.94 (0.35, 2.54) | 0.90 | 1/357 | 1.13 (0.5, 2.57) | 0.76 |
| Has tuberculosis/STI/recent HIV exposure^cd^ | 0/2 |  |  | 0/1 |  |  |
| ^a^Missing data omitted from univariable analysis. ^b^Due to multicollinearity, the characteristic "divorced/separated in prior 12 months" was excluded in multivariable analysis, while the characteristic "separated/divorced marital status" was included. ^c^Characteristics omitted in univariable analysis among men, and ^d^women due to small numbers. Abbreviations: n, number; CI, confidence interval; STI, sexually transmitted infection. | | | | | | |

| **Supplementary Table SII: Multivariable association of sociodemographic and behavioral characteristics with HIV infection among men at the five high-volume facilities** | | | | | |
| --- | --- | --- | --- | --- | --- |
| **Characteristic** | **Full multivariable model** | | **Stepwise multivariable analysis** | | |
|  | **Odds ratio** | **β (95% CI)** | **Odds ratio** | **β (95% CI)** | **Risk score^a^** |
| Ages 25–29, 30–34 and 45–49 years^b^ | 1.82 | 0.6 (0.11, 1.08) | 1.80 | 0.59 (0.1, 1.07) | 6 |
| Ages 35–39 and 40–44 years^b^ | 2.78 | 1.02 (0.51, 1.54) | 2.78 | 1.02 (0.51, 1.54) | 10 |
| Manual/domestic occupation^b^ | 3.57 | 1.27 (0.77, 1.77) | 3.52 | 1.26 (0.76, 1.76) | 13 |
| Trade/sales/service occupation^b^ | 2.63 | 0.97 (0.49, 1.44) | 2.66 | 0.98 (0.5, 1.46) | 10 |
| Married polygamous^b^ | 1.34 | 0.29 (-0.32, 0.9) |  |  |  |
| Widowed^c^ | 4.57 | 1.52 (0.55, 2.49) | 4.40 | 1.48 (0.51, 2.45) | 15 |
| Separated/divorced^b^ | 4.36 | 1.47 (0.71, 2.23) | 4.22 | 1.44 (0.69, 2.19) | 14 |
| >2 sexual partners in prior 12 months^b^ | 1.49 | 0.4 (-0.09, 0.89) | 1.57 | 0.45 (-0.02, 0.92) | 5 |
| New sexual partner in prior 12 months^b^ | 1.46 | 0.38 (-0.24, 0.99) | 1.60 | 0.47 (-0.12, 1.05) | 5 |
| Had sex in exchange for money/favors in prior 12 months^c^ | 1.25 | 0.22 (-0.69, 1.13) |  |  |  |
| Had sex under influence of alcohol/other substance in prior 12 months^c^ | 0.82 | -0.2 (-1.15, 0.75) |  |  |  |
| Circumcised (men only)^b^ | 2.66 | 0.98 (0.62, 1.34) | 2.67 | 0.98 (0.62, 1.35) | 10 |
| Reported treatment for STI in prior 12 months^c^ | 1.99 | 0.69 (-0.4, 1.78) |  |  |  |
| Never been tested for HIV^b^ | 4.62 | 1.53 (1, 2.07) | 4.56 | 1.52 (0.98, 2.05) | 15 |
| ^a^Computed by multiplying the beta regression coefficients by 10 and rounding to the nearest integer. ^b^Variables with significant association with HIV infection in univariable analysis, and included in multivariable analysis. ^c^Variables without significant association with HIV infection in univariable analysis, but included in multivariable analysis based on prior knowledge of association with HIV infection. Abbreviations: β, regression coefficient; CI, confidence interval; STI, sexually transmitted infection. | | | | | |

| **Supplementary Table SIII: Multivariable association of sociodemographic and behavioral characteristics with HIV infection among women at the five high-volume facilities** | | | | | |
| --- | --- | --- | --- | --- | --- |
| **Characteristic** | **Full multivariable model** | | **Stepwise multivariable analysis** | | |
|  | **Odds ratio** | **β (95% CI)** | **Odds ratio** | **β (95% CI)** | **Risk score^a^** |
| Ages 35–39 and 40–44 years^b^ | 1.71 | 0.54 (0.15, 0.92) | 1.71 | 0.54 (0.15, 0.92) | 5 |
| Manual/domestic occupation^b^ | 1.02 | 0.02 (-1.42, 1.45) |  |  |  |
| Trade/sales/service occupation^b^ | 1.94 | 0.66 (0.34, 0.99) | 1.95 | 0.67 (0.34, 1) | 7 |
| Married polygamous^b^ | 3.19 | 1.16 (0.59, 1.73) | 3.19 | 1.16 (0.59, 1.73) | 12 |
| Widowed^b^ | 2.55 | 0.94 (0.38, 1.49) | 2.67 | 0.98 (0.48, 1.49) | 10 |
| Separated/divorced^b^ | 9.61 | 2.26 (1.63, 2.9) | 10.56 | 2.36 (1.81, 2.91) | 24 |
| >2 sexual partners in prior 12 months^b^ | 1.15 | 0.14 (-0.42, 0.7) |  |  |  |
| New sexual partner in prior 12 months^b^ | 3.21 | 1.17 (0.38, 1.96) | 2.76 | 1.02 (0.38, 1.65) | 10 |
| Divorced/separated in prior 12 months^c^ | 1.31 | 0.27 (-0.91, 1.45) |  |  |  |
| Coerced to have sex in prior 12 months^c^ | 1.47 | 0.38 (-0.54, 1.3) |  |  |  |
| Had sex in exchange for money/favors in prior 12 months^c^ | 0.16 | -1.86 (-3.49, -0.22) |  |  |  |
| Had sex under influence of alcohol/other substance in prior 12 months^c^ | 1.41 | 0.34 (-1.2, 1.89) |  |  |  |
| Reported treatment for STI in prior 12 months^b^ | 2.75 | 1.01 (0.04, 1.98) | 2.26 | 0.82 (-0.15, 1.79) | 8 |
| Never been tested for HIV^b^ | 2.89 | 1.06 (0.45, 1.67) | 2.91 | 1.07 (0.46, 1.68) | 11 |
| HIV negative test >12 months prior^b^ | 10.93 | 2.39 (0.2, 4.58) | 10.92 | 2.39 (0.19, 4.59) | 24 |
| ^a^Computed by multiplying the beta regression coefficients by 10 and rounding to the nearest integer. ^b^Variables with significant association with HIV infection in univariable analysis, and included in multivariable analysis. ^c^Variables without significant association with HIV infection in univariable analysis, but included in multivariable analysis based on prior knowledge of association with HIV infection. Abbreviations: β, regression coefficient; CI, confidence interval; STI, sexually transmitted infection. | | | | | |

| **Supplementary Table SIV: Final algorithm risk-score categories for development and validation datasets, stratified by gender** | | | | |
| --- | --- | --- | --- | --- |
| **Men** | | | | |
| **Risk-score categories for algorithm development dataset** | | | | |
| Risk-score category | Number HIV positive/Tested | HIV prevalence, % (95% CI) | % of total HIV positive | % of total tests |
| <12 | 9/2,490 | 0.36% (0.17, 0.69) | 14% | 50% |
| 13–26 | 25/2,044 | 1.22% (0.79, 1.8) | 40% | 41% |
| 27–39 | 19/434 | 4.38% (2.66, 6.75) | 30% | 8% |
| >40 | 10/33 | 30.3% (15.59, 48.71) | 16% | 1% |
| Total | 63/5,001 | 1.26% |  |  |
| **Risk-score categories for algorithm validation dataset** | | | | |
| Risk-score category | Number HIV positive/Tested | HIV prevalence, % (95% CI) | % of total HIV positive | % of total tests |
| <12 | 8/1,765 | 0.45% (0.2, 0.89) | 13% | 43% |
| 13–26 | 35/1,919 | 1.82% (1.27, 2.53) | 55% | 47% |
| 27–39 | 15/379 | 3.96% (2.23, 6.44) | 24% | 9% |
| >40 | 5/35 | 14.29% (4.81, 30.26) | 8% | 1% |
| Total | 63/4,098 | 1.54% |  |  |
| **Women** | | | | |
| **Risk-score categories for algorithm development dataset** | | | | |
| Risk-score category | Number HIV positive/Tested | HIV prevalence, % (95% CI) | % of total HIV positive | % of total tests |
| <7 | 37/6,676 | 0.55% (0.39, 0.76) | 49% | 77% |
| 8–20 | 25/1,767 | 1.41% (0.92, 2.08) | 33% | 20% |
| 21–27 | 8/135 | 5.93% (2.59, 11.34) | 11% | 2% |
| >28 | 5/66 | 7.58% (2.51, 16.8) | 7% | 1% |
| Total | 75/8644 | 0.87% |  |  |
| **Risk-score categories for algorithm validation dataset** | | | | |
| Risk-score category | Number HIV positive/Tested | HIV prevalence, % (95% CI) | % of total HIV positive | % of total tests |
| <7 | 42/4,774 | 0.88% (0.63, 1.19) | 47% | 76% |
| 8–20 | 30/1,302 | 2.3% (1.56, 3.27) | 34% | 21% |
| 21–27 | 10/118 | 8.47% (4.14, 15.03) | 11% | 2% |
| >28 | 7/61 | 11.48% (4.74, 22.22) | 8% | 1% |
| Total | 89/6,255 | 1.42% |  |  |
